# Gender Differences in Prognosis and Risk Stratification of Brugada Syndrome: A Pooled Analysis of 4,140 Patients From 24 Clinical Trials

**DOI:** 10.3389/fphys.2018.01127

**Published:** 2018-08-22

**Authors:** Mengchen Yuan, Chao Tian, Xinye Li, Xinyu Yang, Xiaofeng Wang, Yihan Yang, Nian Liu, Kengo F. Kusano, Hector Barajas-Martinez, Dan Hu, Hongcai Shang, Yonghong Gao, Yanwei Xing

**Affiliations:** ^1^Guang'anmen Hospital, Chinese Academy of Chinese Medical Sciences, Beijing, China; ^2^Key Laboratory of Chinese Internal Medicine of Ministry of Education and Beijing, Dongzhimen Hospital Affiliated to Beijing University of Chinese Medicine, Beijing, China; ^3^Department of Cardiology, Beijing An Zhen Hospital of the Capital University of Medical Sciences, Beijing, China; ^4^Division of Arrhythmia and Electrophysiology, Department of Cardiovascular Medicine, National Cerebral and Cardiovascular Center, Suita, Osaka, Japan; ^5^Masonic Medical Research Laboratory, Utica, NY, United States; ^6^Hubei Key Laboratory of Cardiology, Department of Cardiology and Cardiovascular Research Institute, Renmin Hospital of Wuhan University, Wuhan, China

**Keywords:** Brugada syndrome, gender difference, electrophysiological study, prognosis, risk stratification

## Abstract

**Background:** Male gender has been consistently shown to be a risk factor for a greater number of arrhythmic events in patients with Brugada Syndrome (BrS). However, there have been no large-scale comprehensive pooled analyses to statistically and systematically verify this association. Therefore, we conducted a pooled analysis on gender differences in prognosis and risk stratification of BrS with a largest sample capacity at present.

**Methods:** We searched PubMed, Embase, Medline, Cochrane Library databases, Chinese National Knowledge Infrastructure, and Wanfang Data for relevant studies published from 2002 to 2017. The prognosis and risk stratification of BrS and risk factors were then investigated and evaluated according to gender.

**Results:** Twenty-four eligible studies involving 4,140 patients were included in the analysis. Male patients (78.1%) had a higher risk of arrhythmic events than female patients (95% confidence interval: 1.46–2.91, *P* < 0.0001). Among the male population, there were statistical differences between symptomatic patients and asymptomatic patients (95% CI: 2.63–7.86, *P* < 0.00001), but in the female population, no statistical differences were found. In the female subgroup, electrophysiological study (EPS) positive patients had a tendency toward a higher risk of arrhythmic events than EPS-negative patients (95% CI: 0.93–29.77, *P* = 0.06).

**Conclusions:** Male patients are at a higher risk of arrhythmic events than female patients. Within the male population, symptomatic patients have a significantly higher risk profile compared to asymptomatic patients, but no such differences are evident within the female population. Consequently, in the female population, the risk of asymptomatic patterns cannot be underestimated.

## Introduction

Brugada syndrome (BrS) is an inherited arrhythmic disorder generally characterized by a distinct electrocardiogram (ECG) pattern: the presence of ST-segment elevation in the right precordial leads (V1–V3), which may carry an increased risk of sudden cardiac death (SCD) due to malignant ventricular arrhythmias (Bayés et al., 2012). That typical “syndrome” was firstly presented by Nava et al. in 1988 at the National Congress of Italian Cardiologists, which subsequently named by Brugada brothers in 1992 (Martini et al., 1988; Nava et al., 1988; Brugada and Brugada, 1992). In current common consensus, BrS was described as a functional abnormality of repolarization, but theory proposed by Nava et al. believed that the true syndrome is not only a primary electrical disease performed particular ECG but a conduction disturbance at the right ventricular outflow tract (RVOT) related to clinical events (Martini and Nava, 2004; Marras et al., 2009). Recent focal therapeutic radiofrequency ablation (RFA) strategy indirectly proved the theory (Brugada et al., 2015). According to the expert consensus in 2013, patients with Brugada type 1 ECG induced by Class I antiarrhythmic drugs are included (Priori et al., 2013). Type 1 ECG induced by drug may occur false positive Brugada (Konigstein et al., 2016; Mizusawa et al., 2016).

Male sex has consistently been shown to be associated with a higher risk of arrhythmic events (Benito et al., 2008; Priori et al., 2013). However, the lack of large-scale samples and systematic comprehensive analysis have contributed to weak conformance and statistical power. In addition, there have been no comprehensive pooled analyses examining prognosis and risk stratification for BrS. Several clinical variables are considered to be potentially associated with worse outcome in patients with BrS. Electrophysiological study (EPS) might be the most controversial factor, and there remains no consensus on whether its inducibility is valuable in predicting outcome (Priori et al., 2002, 2012; Eckardt et al., 2005). Large registries have consistently shown that patients with spontaneous type 1 ECG have a high risk of cardiac arrhythmic events at follow-up (Brugada et al., 2002, 2004, 2005). The presence of symptoms is a significant predictor of arrhythmias (Priori et al., 2002). *SCN5A* mutation and recent positive family history of SCD have debatable feasibility as risk markers (Kanda et al., 2002). Lack of examination for documented auricular fibrillation (AF) status might lead to new agitation. However, the gender differences between these variables and whether men and women experience disparate outcomes remain indeterminate. Variables differing between the sexes, and how these manifests in certain sex groups, remain to be elucidated.

Therefore, based on a largest sample capacity of 4,140 patients from 24 clinical trials at present, we conducted a comprehensive pooled analysis of gender differences, including the following aspects: risk of arrhythmic events, EPS status, family history of SCD, spontaneous type 1 ECG pattern, *SCN5A* mutation, diagnosis status, and documented AF status.

## Methods

### Search strategy

**Figure 1:**
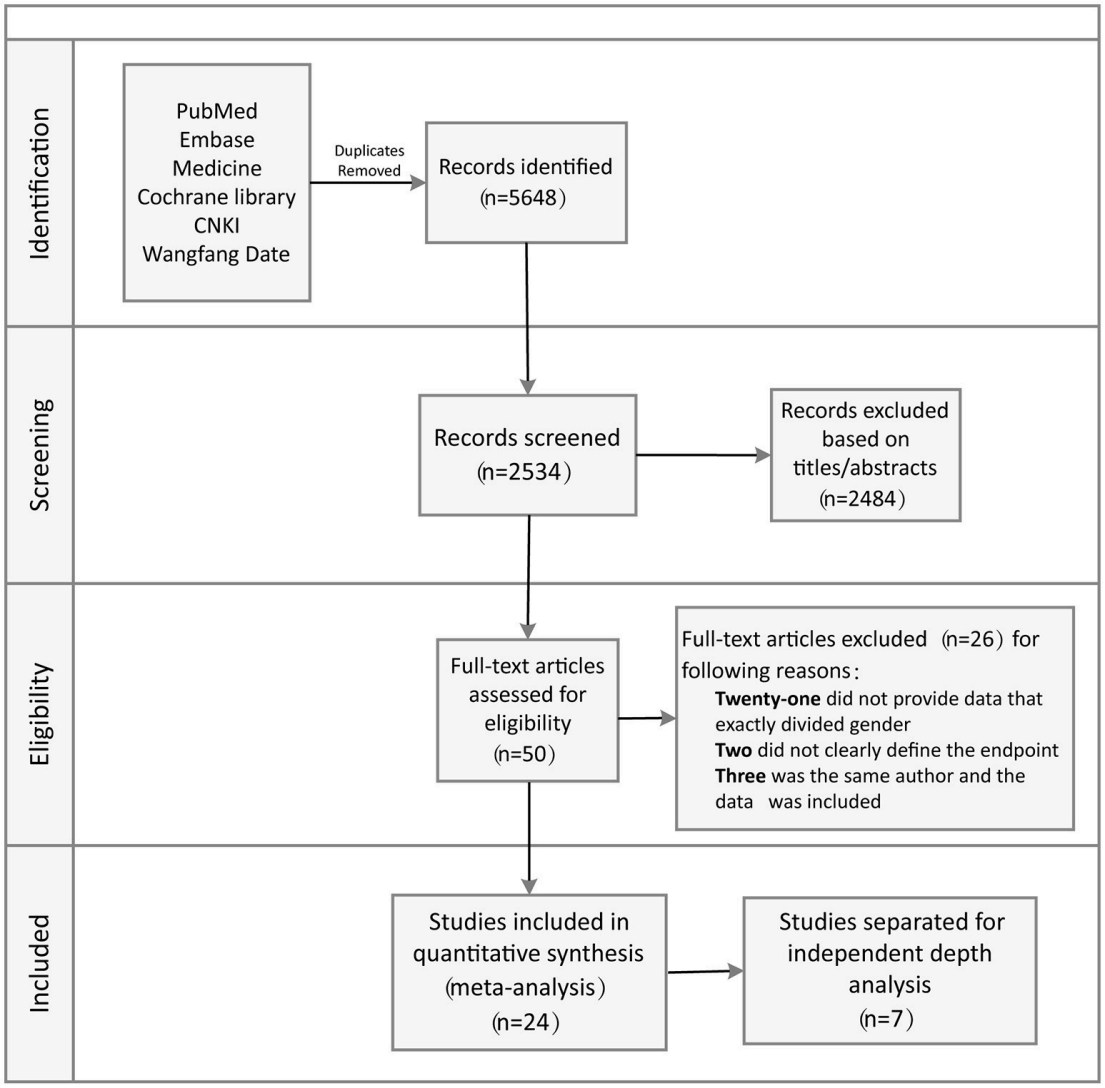
Flow diagram of data search and study selection.

A comprehensive literature search of relevant studies published in PubMed, Embase, Medline, Cochrane Library databases, Chinese National Knowledge Infrastructure, and Wanfang Data was performed by two reviewers independently and systematically. We searched relevant published studies from 2002 to 2017 using the keywords: “Brugada” and “syndrome” or “Brugada syndrome” and “risk stratification.” The titles, abstracts, and reference lists of all articles were carefully reviewed for potential and additional publications regarding this topic. Full text assessment of potential relevant studies was conducted for compliance with the inclusion criteria and to prevent duplication of data by the same group of authors (Figure 1).

### Inclusion criteria

All studies had to meet following criteria for inclusion: (a) full-text English language studies published in peer-reviewed journals; (b) prospective or retrospective observational study design; (c) follow-up duration sufficiently long to detect arrhythmic events; (d) information included regarding clearly defined endpoint events (appropriate shocks, ventricular fibrillation/ventricular tachycardia, and SCD); (e) risk ratio (RR), hazard ratio (HR), odds ratio (OR), corresponding 95% confidence intervals (CIs), or necessary raw data were reported.

### Data collection

Twenty-four studies (Kanda et al., 2002; Masaki et al., 2002; Priori et al., 2002, 2012; Slim et al., 2003; Mok et al., 2004; Furushima et al., 2005; Kharazi et al., 2007; Ohkubo et al., 2007; Sarkozy et al., 2007, 2011; Benito et al., 2008; Morita et al., 2008; Sacher et al., 2008, 2013; Giustetto et al., 2009; Kamakura et al., 2009; Schukro et al., 2010; Son et al., 2013; Tokioka et al., 2014; Conte et al., 2015; Sieira et al., 2015; Andorin et al., 2016; Calò et al., 2016; de Asmundis et al., 2017; Yamagata et al., 2017) consisting of 4,140 BrS patients were ultimately included in the study analysis. The extracted data elements for the analysis included: surname of first author, publication year, origin of the studied population, type of study, study design, study population, mean duration follow-up, endpoint events, quality score (Table 1); sample size, participants' age and sex, number of subjects with history of SCD or syncope, family history of SCD, spontaneous type 1 ECG pattern, detailed information regarding EPS, positive/negative *SCN5A* gene mutation, presence of AF, fragmented QRS (f-QRS), and early repolarization (ER) (Table 2).

**Table 1:** Study characteristics of 24 studies included in pooled analysis.

**Investigator**	**Location**	**Type of study**	**Study of design**	**Study population**	**Mean follow-up**	**Endpoint**	**Quality score**
Kanda et al., 2002	Japan	SC	PS	Patients with symptomatic Brugada syndrome	38 months	Apparent syncope, SCD/VF documented in the storage memory of the ICD	16
Masaki et al., 2002	Japan	SC	PS	Patients identified with an ECG pattern consisting of right bundle branch block with ST elevation in leads V 1–V 3	36 ± 24 months	Sudden death	16
Priori et al., 2002	Italy	MC	PS	Patients with presence of ST-segment elevation ≥2 mm in leads V 1–V 3 at baseline	34 ± 44 months	Documented cardiac arrest	16
Mok et al., 2004	Hong Kong	MC	PS	Patients with type 1 Brugada ECGs	25.8 ± 10.9 months	Syncope/syncopal ventricular arrhythmia/sudden death/appropriate ICD shock	20
Furushima et al., 2005	Japan	SC	PS	Patients with Brugada syndrome	33 ± 16 months	VT/VF/completion of the programmed stimulation protocol	16
Kharazi et al., 2007	IRAN	SC	PS	Patients with Brugada syndrome underwent ICD implantation	27.83 ± 11.25 months	VF/VT/completion of EPS protocol	16
Ohkubo et al., 2007	Japan	SC	PS	Patients with Brugada syndrome	47.1 ± 33.7 months	Sudden cardiac death	16
Sarkozy et al., 2007	Belgium/ Holland	SC	PS	Patients underwent an ICD implantation with the diagnosis of BS	47.5 months	Appropriate shocks	16
Benito et al., 2008	Spain	MC	PS	Patients with Brugada syndrome	58 ± 48 months	SCD/documented VF	16
Morita et al., 2008	Japan	MC	PS	Patients with Brugada-type ECG	43 ± 25 months	SCD/VF/non-cardiac death	20
Sacher et al., 2008	Europe	MC	PS	Patients with a type1 Brugada pattern on at least one baseline ECG/ after provocation with a class I antiarrhythmic drug	4 ± 3 years	Appropriate shocks	16
Giustetto et al., 2009	Italy	MC	PS	Patients with Brugada-type ECG	30 ± 21 months	Arrhythmic events (sudden death/VF)	16
Schukro et al., 2010	Austria	MC	PS	Patients with characteristic ECG either at rest or after provocation with Ajmaline	60.7± 44.2 months	VF	16
Sarkozy et al., 2011	Belgium/ Spain	SC	PS	Patients with diagnostic coved type I ECG	59 months	Sudden death	16
Priori et al., 2012	Italy	MC	PS	Patients with type 1 ECGs, without history of cardiac arrest	36 ± 8 months	The occurrence of VF or appropriate ICD interventions	16
Sacher et al., 2013	France	SC	PS	Patients with type 1 Brugada ECGs with implantable cardioverter-defibrillator	77 ± 42 months	Aborted sudden cardiac arrest/syncope	15
Son et al., 2013	Korea	MC	PS	Patients with BrS and underwent ICD therapy	59 ± 46 months	Appropriate shocks	16
Tokioka et al., 2014	Japan	SC	RS	Patients with a Brugada-type ECG	45.1 ± 44.3 months	VF/SCD	16
Conte et al., 2015	Belgium	SC	RS	Presenting with spontaneous or drug-induced Brugada type 1 ECG and underwent ICD institution	83.8 ± 57.3 months	Appropriate shocks	16
Sieira et al., 2015	Belgium	SC	PS	Patients with spontaneous or drug-induced Brugada type I ECG	73.2 ± 58.9 months	SCD/ICD shock	16
Andorin et al., 2016	Europe	MC	PS	Patients with Brugada ECG under 19 years of age	54 months	Sudden death/documented VT or VF/appropriate ICD shock	16
Calò et al., 2016	Italy	MC	PS	Patients with spontaneous type 1 BrS ECG phenotype	48 ± 38.6 months	VF/SCD	16
de Asmundis et al., 2017	Belgium	SC	PS	Patients with type 1 Brugada ECG pattern	10.1 ± 4.6 years	SCD/ICD shock	16
Yamagata et al., 2017	Japan	MC	PS	Patients with type 1 Brugada ECG pattern	72 months	Documented atrial fibrillation/appropriate ICD interventions	16

*BrS, Brugada syndrome; ECG, electrocardiogram; ICD, implantable cardioverter defibrillator; MC, multi-center study; PS, prospective study; RS, retrospective study; SC, single center study; SCD, sudden cardiac death; VF, ventricular fibrillation; VT, ventricular tachycardia; MINOR, methodological index for non-randomized studies*.

**Table 2:** Clinical characteristics of study patients.

	**Kanda et al., 2002**	**Masaki et al., 2002**	**Priori et al., 2002**	**Mok et al., 2004**	**Furushima et al., 2005**	**Kharazi et al., 2007**	**Ohkubo et al., 2007**	**Sarkozy et al., 2007**
Total Patients, *n*	34	13	200	50	24	12	34	47
Age (years)	44 ± 12	52.4 ± 11.0	41 ± 18	53	61 ± 16	46.5 ± 11.8	52 ± 13	44.5 ± 15
Events, *n* (%)	15 (44)	1 (7.8)	22 (11)	6 (12)	1 (4.2)	2 (17)	1 (29)	7 (15)
Male, *n*	33	12	152	47	23	11	33	35
Events, *n* (%)	15 (45)	1 (8.3)	20 (13)	6 (13)	1 (4.3)	2 (18)	1 (30)	7 (20)
Female, *n*	1	1	48	3	1	1	1	12
Events, *n* (%)	0 (0)	0 (0)	2 (4.2)	0 (0)	0 (0)	0 (0)	0 (0)	0 (0)
History of SCD, *n* (%)	23 ()	1 (7.8)	22 (11)	8 (15)	7 (29)	2 (17)	2 (59)	NA
History of syncope, *n* (%)	34 (68)	2 (15)	34 (17)	12 (24)	8 (33)	7 (58)	9 (26)	26 (55)
Asymptomatic. *n* (%)	0 (0)	10 (78)	NA	30 (60)	9 (37.5)	3 (25)	23 (68)	NA
Family history of SCD, *n* (%)	4 (12)	NA	NA	7 (14)	NA	2 (17)	3 (8.8)	26 (55)
Spontaneous type1 ECG, *n* (%)	NA	9 (69)	NA	43 (86)	NA	NA	12 (35)	23 (49)
Events, *n* (%)	NA	1 (11)	NA	17 (40)	NA	NA	1 (83)	7 (30)
Non-spontaneous type1 ECG, *n* (%)	NA	4 (31)	NA	7 (14)	NA	NA	22 (65)	NA
Events, *n* (%)	NA	0 (0)	NA	3 (43)	NA	NA	0 (0)	NA
Underwent EPS, *n* (%)	34 (100)	13 (100)	29 (14.5)	30 (60)	22 (92)	4 (33)	34 (100)	46 (98)
EPS+, (*n*)	22 (65)	8 (62)	0 (0)	19 (63)	20 (91)	4 (100)	28 (82)	38 (83)
EPS–, (*n*)	12 (35)	5 (38)	29 (100)	11 (37)	2 (9)	0 (0)	6 (18)	8 (17)
AF (+), *n* (%)	NA	NA	NA	NA	NA	NA	NA	NA
Underwent DNA testing, *n* (%)	NA	NA	NA	36 (72)	NA	NA	NA	NA
*SCN5A* (+)*, n* (%)	NA	NA	NA	5 (14)	NA	NA	NA	NA
Symptomatic, *n* (%)	NA	NA	NA	2 (40)	NA	NA	NA	NA
*SCN5A* (–)*, n* (%)	NA	NA	NA	31 (86)	NA	NA	NA	NA
Symptomatic, *n* (%)	NA	NA	NA	18 (58)	NA	NA	NA	NA
f-QRS (+)*, n* (%)	NA	NA	NA	NA	NA	NA	NA	NA
f-QRS (–)*, n* (%)	NA	NA	NA	NA	NA	NA	NA	NA
ER (+)*, n* (%)	NA	NA	NA	NA	NA	NA	NA	NA
ER (–)*, n* (%)	NA	NA	NA	NA	NA	NA	NA	NA
	**Benito et al.**, **2008**	**Morita et al.**, **2008**	**Sacher et al.**, **2008**	**Giustetto et al.**, **2009**	**Schukro et al.**, **2010**	**Sarkozy et al.**, **2011**	**Priori et al.**, **2012**	**Sacher et al.**, **2013**
Total Patients, *n*	384	115	58	166	26	280	308	378
Age (years)	45.9 ± 22	48 ± 12	47 ± 11	45+14	43.2 ± 11.6	41+18	47 ± 12	46 ± 13
Events, *n* (%)	34 (8.9)	18 (16)	31 (53)	9 (5.4)	2 (7.7)	18 ()	14 (4.5)	46 (12)
Male, *n*	272	113	50	138	20	168	257	310
Events, *n* (%)	31 (11)	18 (16)	25 (50)	9 (6.5)	2 (10)	16 ()	11 (4.3)	42 (14)
Female, *n*	112	2	8	28	6	112	41	68
Events, *n* (%)	3 (2.5)	0 (0)	6 (75)	0 (0)	0 (0)	2 ()	3 (7.3)	4 (5.9)
History of SCD, *n* (%)	NA	NA	36 (62)	5 (3)	4 (15)	14 ()	NA	31 (8.2)
History of syncope, *n* (%)	NA	NA	NA	58 (35)	7 (27)	68 ()	65 (21)	181 (48)
Asymptomatic, *n* (%)	301 (78)	NA	NA	103 (62)	15 (58)	169 ()	243 (80)	166 (44)
Family history of SCD, *n* (%)	NA	NA	NA	39 (23)	NA	149 ()	NA	111 (29)
Spontaneous type1 ECG, *n* (%)	154 (40)	NA	NA	72 (43)	11 (42)	65 ()	171 (56)	226 (60)
Events, *n* (%)	23 (15)	NA	NA	5 (7)	2 (18)	12 ()	13 (7.6)	35 (15)
Non-spontaneous type1 ECG, *n* (%)	230 (60)	NA	NA	94 (57)	15 (58)	215 ()	NA	152 (60)
Events, *n* (%)	11 (4.8)	NA	NA	4 (4.3)	0 (0)	6 ()	NA	11 (7.2)
Underwent EPS, *n* (%)	350 (91)	NA	NA	135 (81)	14 (54)	NA	238 (77)	310 (82)
EPS+, (*n*)	95 (27)	NA	NA	46 (34)	8 (57)	NA	61 (26)	228 (74)
EPS–, (*n*)	255 (73)	NA	NA	89 (66)	6 (43)	NA	177 (74)	82 (26)
AF (+), *n* (%)	40 (10)	NA	NA	NA	NA	NA	NA	32 (8.5)
Underwent DNA testing, *n* (%)	350 (91)	NA	NA	NA	NA	NA	123 (40)	160 (43)
*SCN5A* (+)*, n* (%)	95 (27)	NA	NA	NA	NA	NA	24 (20)	41 (26)
Symptomatic, *n* (%)	21 (22)	NA	NA	NA	NA	NA	3 (12.5)	6 (15)
*SCN5A* (–), *n* (%)	255 (73)	NA	NA	NA	NA	NA	99 (80)	119 (74)
Symptomatic, *n* (%)	8 (3.1)	NA	NA	NA	NA	NA	6 (14)	16 (13)
f-QRS (+)*, n* (%)	NA	50 (43)	NA	NA	NA	NA	25 (8)	NA
f-QRS (–)*, n* (%)	NA	65 (57)	NA	NA	NA	NA	283 (92)	NA
ER (+)*, n* (%)	NA	NA	NA	NA	NA	NA	NA	NA
ER (–)*, n* (%)	NA	NA	NA	NA	NA	NA	NA	NA
	**Son et al.**, **2013**	**Tokioka et al.**, **2014**	**Conte et al.**, **2015**	**Sieira et al.**, **2015**	**Andorin et al.**, **2016**	**Calò et al.**, **2016**	**de Asmundis et al.**, **2017**	**Yamagata et al.**, **2017**
Total Patients, *n*	69	246	176	363	106	347	289	415
Age (years)	46.2 ± 13.5	47.6 ± 13.6	43.3 ± 16.8	40.9 ± 17.2	11.1 ± 5.7	45 ± 13.1	45 ± 16	46 ± 14
Events, *n* (%)	19 (28)	24 (9.8)	28 (16)	9 (2.5)	10 (9.4)	32 (9.2)	29 (10)	62 (15)
Male, *n*	68	236	118	200	58	272	203	403
Events, *n* (%)	19 (28)	23 (9.7)	24 (20)	7 (3.5)	6 (10)	28 (10)	24 (12)	62 (15)
Female, *n*	1	10	58	163	48	75	86	12
Events, *n* (%)	0 (0)	1 (10)	4 (6.9)	2 (1.2)	4 (8.3)	4 (5.3)	5 (5.8)	0 (0)
History of SCD, *n* (%)	38 (55)	13 (5.3)	25 (15)	NA	NA	0 (0)	17 (5.9)	88 (21)
History of syncope, *n* (%)	17 (25)	40 (16)	105 (60)	NA	NA	14 (4)	103 (36)	99 (24)
Asymptomatic, *n* (%)	14 (20)	NA	46 (26)	NA	85 (80)	316 (91)	NA	228 (55)
Family history of SCD, *n* (%)	NA	69 (28)	NA	182 (50)	46 (43)	71 (20)	99 (34)	64 (15)
Spontaneous type1 ECG, *n* (%)	46 (67)	156 (63)	37 (21)	41 (11)	36 (34)	347 (100)	79 (27)	299 (72)
Events, *n* (%)	12 (26)	22 (14)	16 (43)	3 (7.3)	8 (22)	32 (9.2)	19 (24)	48 (16)
Non-spontaneous type1 ECG, *n* (%)	23 (33)	90 (37)	139 (79)	322 (89)	70 (66)	0 (0)	210 (73)	116 (28)
Events, *n* (%)	7 (30)	2 (2.2)	12 (8.6)	6 (1.9)	2 (2.9)	0 (0)	10 (4.8)	14 (12)
Underwent EPS, *n* (%)	NA	155 (63)	NA	321 (88)	NA	186 (54)	NA	339 (82)
EPS+, (*n*)	NA	71 (46)	NA	32 (10)	NA	77 (41)	NA	191 (56)
EPS–, (*n*)	NA	84 (54)	NA	289 (90)	NA	109 (59)	NA	148 (44)
AF (+)*, n* (%)	NA	44 (18)	NA	NA	NA	NA	31 (11)	64 (15)
Underwent DNA testing, *n* (%)	NA	123 (50)	NA	NA	75 (71)	107 (31)	37 (13)	415 (100)
*SCN5A* (+)*, n* (%)	NA	17 (14)	NA	NA	58 (77)	32 (30)	32 (86)	60 (14)
Symptomatic, *n* (%)	NA	4 (24)	NA	NA	9 (16)	2 (6)	5 (14)	13 (38)
*SCN5*A (-)*, n* (%)	NA	106 (86)	NA	NA	17 (23)	75 (70)	NA	355 (86)
Symptomatic, *n* (%)	NA	19 (18)	NA	NA	0 (0)	10 (13)	NA	49 (14)
f-QRS (+), *n* (%)	NA	78 (32)	NA	NA	NA	85 (24)	50 (17)	NA
f-QRS (–), *n* (%)	NA	168 (68)	NA	NA	NA	262 (76)	239 (83)	NA
ER (+), *n* (%)	NA	25 (10)	NA	NA	NA	30 (9)	NA	NA
ER (–), *n* (%)	NA	221 (90)	NA	NA	NA	317 (91)	NA	NA

*ECG, electrocardiogram; SCD, sudden cardiac death; VT, ventricular tachycardia; VF, ventricular fibrillation; f-QRS, fragmented QRS; NA, not available; n, number; EPS, electrophysiological study; AF, auricular fibrillation; ER, early repolarization*.

Upon sending e-mails to the principal authors of identified studies to request data sharing with a standardized form and definitions, we received original data for two of the studies (Sacher et al., 2013; Tokioka et al., 2014). Some of the data could not be found in the articles because the original data might be different from that published, owing to additional patients and longer follow-up times.

### Quality assessment

The Methodological Index for Non-Randomized Studies (MINORS) (Slim et al., 2003) was used to assess the quality of all included studies. The maximum value with this index is 24 points, with each item scored from 0 to 2 on the following aspects: (a) a clearly stated aim; (b) inclusion of consecutive patients; (c) prospective collection of data; (d) endpoints appropriate to the aim of the study; (e) unbiased assessment of the study endpoint; (f) follow-up period appropriate to the aim of the study; (g) loss to follow up < 5%; and (h) prospective calculation of the study size. Both reviewers independently scored the included publications, then used the average MINORS score for final assessment. Based on MINORS scores of <16 and ≥16 points, studies were defined to be low-quality and high-quality studies, respectively.

### Statistical analysis

We estimated heterogeneity between studies using I^2^, which is derived from the standard chi-square test to represent the variability in effect produced by heterogeneity. An I^2^ >50% was indicative of significant statistical heterogeneity. We extracted and analyzed all the multivariate adjusted OR with 95% CI for each study. Pooled OR were calculated using the M-H random-effects model and fixed-effects model to take into account within-study and between-study variance. Sensitivity analyses were conducted to evaluate the significance of the final results. We also performed subgroup analysis based on gender (positive vs. negative), EPS status (male vs. female), family history of SCD (male vs. female), spontaneous type 1 ECG (male vs. female), *SCN5A* (male vs. female), status at diagnosis (male vs. female), and documented AF status (male vs. female), and the OR was also calculated. Publication bias was assessed by means of the funnel plot. Statistical significance was defined as a *P*-value ≤ 0.05. All analyses were performed using Review Manager, version 5.0.12 (Revman; The Cochrane Collaboration, Oxford, U.K.).

## Results

### Study selection

The systematic review of the literature yielded a total of 5,648 potentially relevant studies with our search criteria. After screening of the titles and abstracts, 2,534 studies were excluded, leaving 50 for full-text assessment. Twenty-six duplicate studies were excluded, while 21 did not provide clear data pertaining to sex-related differences. Two studies did not clearly define the endpoint, while three had the same author with data included. Eventually, 24 of the original qualifying studies from the databases were included. Seven of the 24 studies were separated for independent depth analysis (Figure 1).

### Male and female

**Figure 2:**
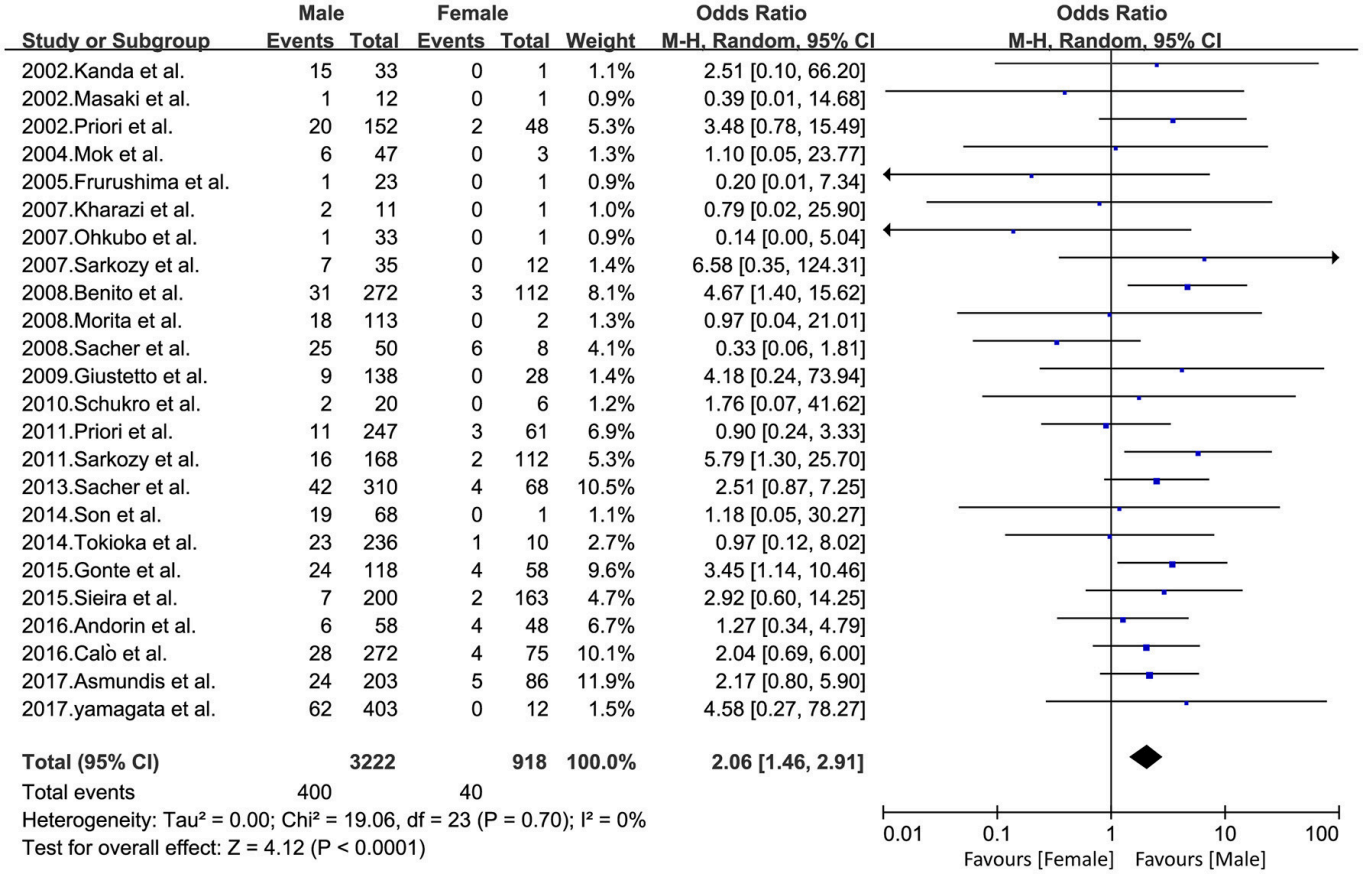
Odds radio for the occurrence of arrhythmic events during follow-up depending on the presence of gender.

Overall, among 4,140 patients with BrS, 3,222 male patients (event rate 12.4%) and 918 female patients (event rate 4.4%) were included, because BrS is a male predominance syndrome (Priori et al., 2013). All 24 studies were included in this pooled gender analysis. An increased risk of arrhythmic events was observed in the male population compared to the female population (OR 2.06, 95% CI: 1.46–2.91, *P* < 0.0001; heterogeneity: *P* = 0.70, I^2^ = 0%, Figure 2). The calculations showed a statistically significant difference between the two groups. Males had a higher risk of arrhythmia compared to females. At the same time, we conducted sensitivity analysis, excluding any set of data that would have no effect on the results.

### EPS group

**Figure 3:**
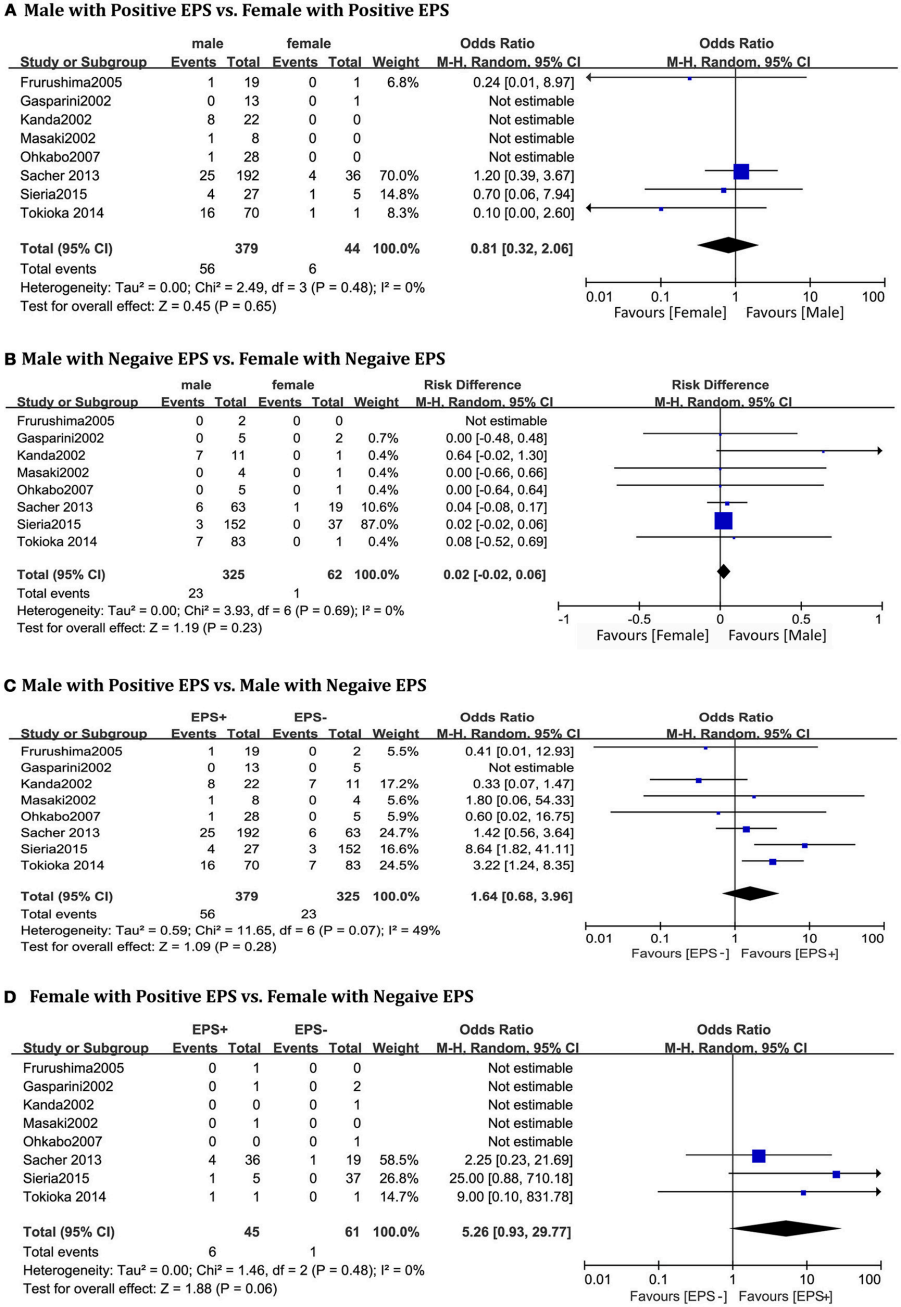
Odds radio for the occurrence of arrhythmic events during follow-up depending on EPS pattern subgroups. **(A)** Prognosis of male and female in positive EPS subgroup, **(B)** Prognosis of male and female in negative EPS subgroup, **(C)** Prognosis of positive EPS and negative EPS in male subgroup, **(D)** Prognosis of positive EPS and negative EPS in female subgroup.

A total of 810 patients (men = 704) from eight studies (Kanda et al., 2002; Masaki et al., 2002; Furushima et al., 2005; Ohkubo et al., 2007; Priori et al., 2012; Sacher et al., 2013; Tokioka et al., 2014; Sieira et al., 2015) were included in this group. In the EPS-positive subgroup, no significant gender differences related to cardiac events were found between males and females(OR 0.81, 95% CI: 0.32–2.06, *P* = 0.65; heterogeneity: *P* = 0.48, I^2^ = 0 %, Figure 3A). The result was the same in the EPS-negative subgroup (OR 0.02, 95% CI: −0.02–0.06, *P* = 0.23; heterogeneity: *P* = 0.69, I^2^ = 0%, Figure 3B). In the male subgroup, there was also no statistical difference between EPS-positive patients and EPS-negative patients (OR 1.64, 95% CI: 0.68–3.96, *P* = 0.28; heterogeneity: *P* = 0.07, I^2^ = 49%, Figure 3C). However, in the female subgroup, EPS-positive patients had a tendency toward a higher risk of arrhythmic events (OR 5.26, 95% CI: 0.93–29.77, *P* = 0.06; heterogeneity: *P* = 0.48, I^2^ = 0%, Figure 3D).

### Family history of SCD

**Figure 4:**
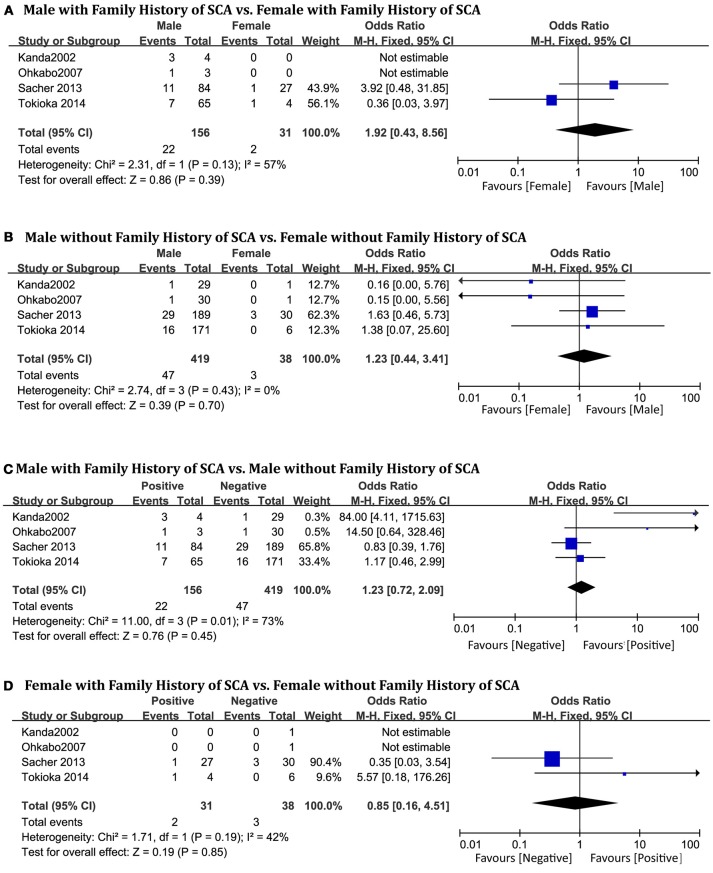
Odds radio for the occurrence of arrhythmic events during follow-up depending on family history of SCA subgroups. **(A)** Prognosis of male and female in positive family history of SCA subgroup, **(B)** Prognosis of male and female in negative family history of SCA subgroup, **(C)** Prognosis of positive family history of SCA and negative family history of SCA in male subgroup, **(D)** Prognosis of positive family history of SCA and negative family history of SCA in female subgroup.

Four studies (Shaowen Liu and Ole Kongstad, 2001; Ohkubo et al., 2007; Bayés et al., 2012; Tokioka et al., 2014), consisting of 634 patients (men = 565) were eligible for this pooled analysis. We did not find significant gender differences in relation to family history of SCD in patients with a positive history (OR 1.92, 95% CI: 0.43–8.56, *P* = 0.39; heterogeneity: *P* = 0.13, I^2^ = 57%, Figure 4A) or in those with a negative history (OR 1.23, 95% CI: 0.44–3.41, *P* = 0.70; heterogeneity: *P* = 0.43, I^2^ = 0%, Figure 4B). There were also no significance differences within the male subgroup (OR 1.23, 95% CI: 0.72–2.09, *P* = 0.45; heterogeneity: *P* = 0.02, I^2^ = 73%, Figure 4C), or within the female subgroup (OR 0.85, 95% CI: 0.16–4.51, *P* = 0.85; heterogeneity: *P* = 0.19, I^2^ = 42%, Figure 4D).

### Spontaneous type 1 ECG pattern

**Figure 5:**
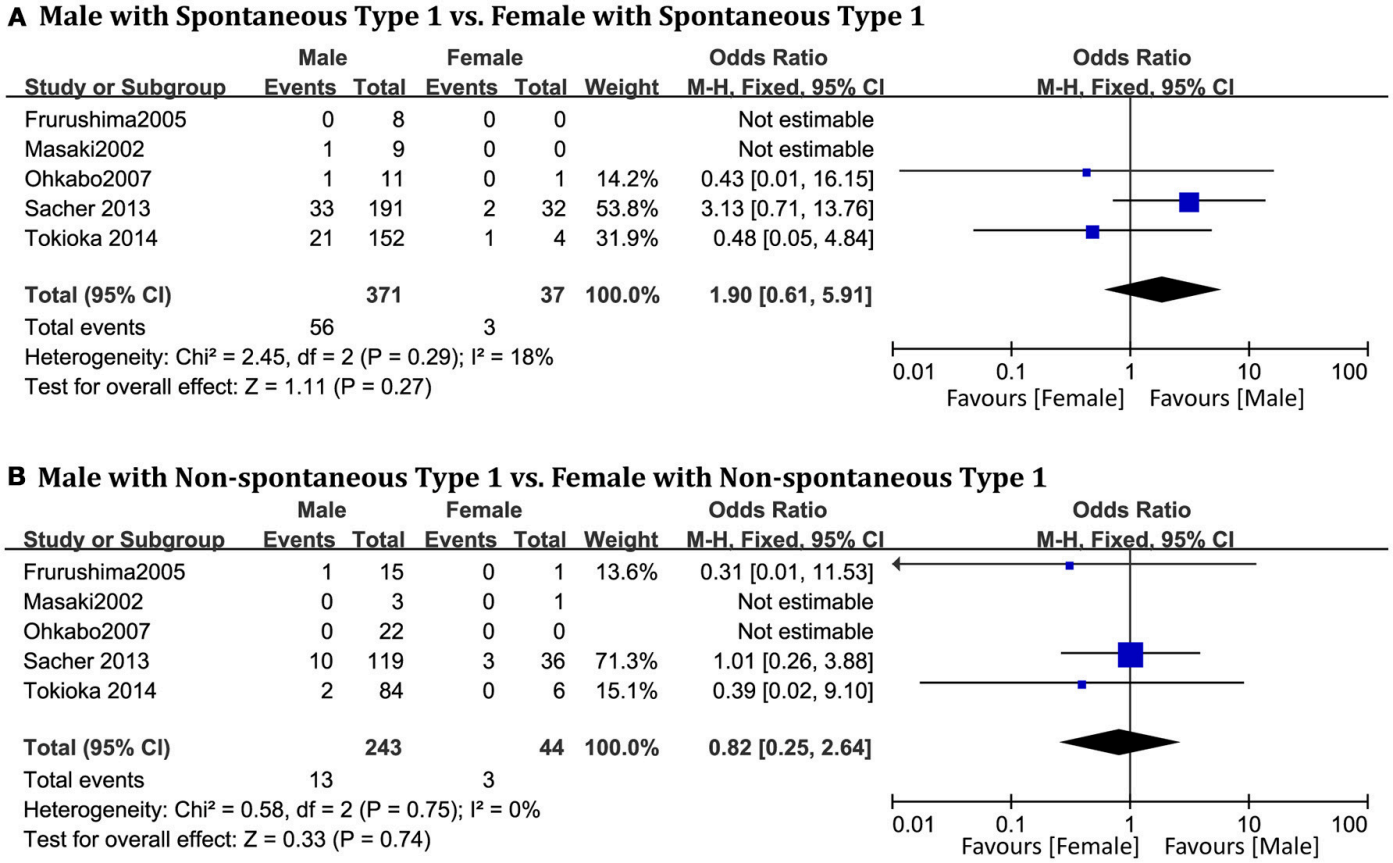
Odds radio for the occurrence of arrhythmic events during follow-up depending on spontaneous type 1 pattern subgroups. **(A)** Prognosis of male and female in spontaneous type 1 subgroup, **(B)** Prognosis of male and female in non-spontaneous type 1 subgroup.

A total of 694 patients (men = 420) from five studies (Masaki et al., 2002; Furushima et al., 2005; Ohkubo et al., 2007; Sacher et al., 2013; Tokioka et al., 2014) were included. No statistically significant sex-related differences were observed in the spontaneous type 1 BrS subgroup (OR 1.90, 95% CI: 0.61–5.91, *P* = 0.27; heterogeneity: *P* = 0.29, I^2^ = 18%, Figure 5A). In the non-spontaneous type 1 ECG subgroup, there was also no statistical difference between men and women (OR 0.82, 95% CI: 0.25–2.64, *P* = 0.74; heterogeneity: *P* = 0.75, I^2^ = 0%, Figure 5B).

### SCN5A

**Figure 6:**
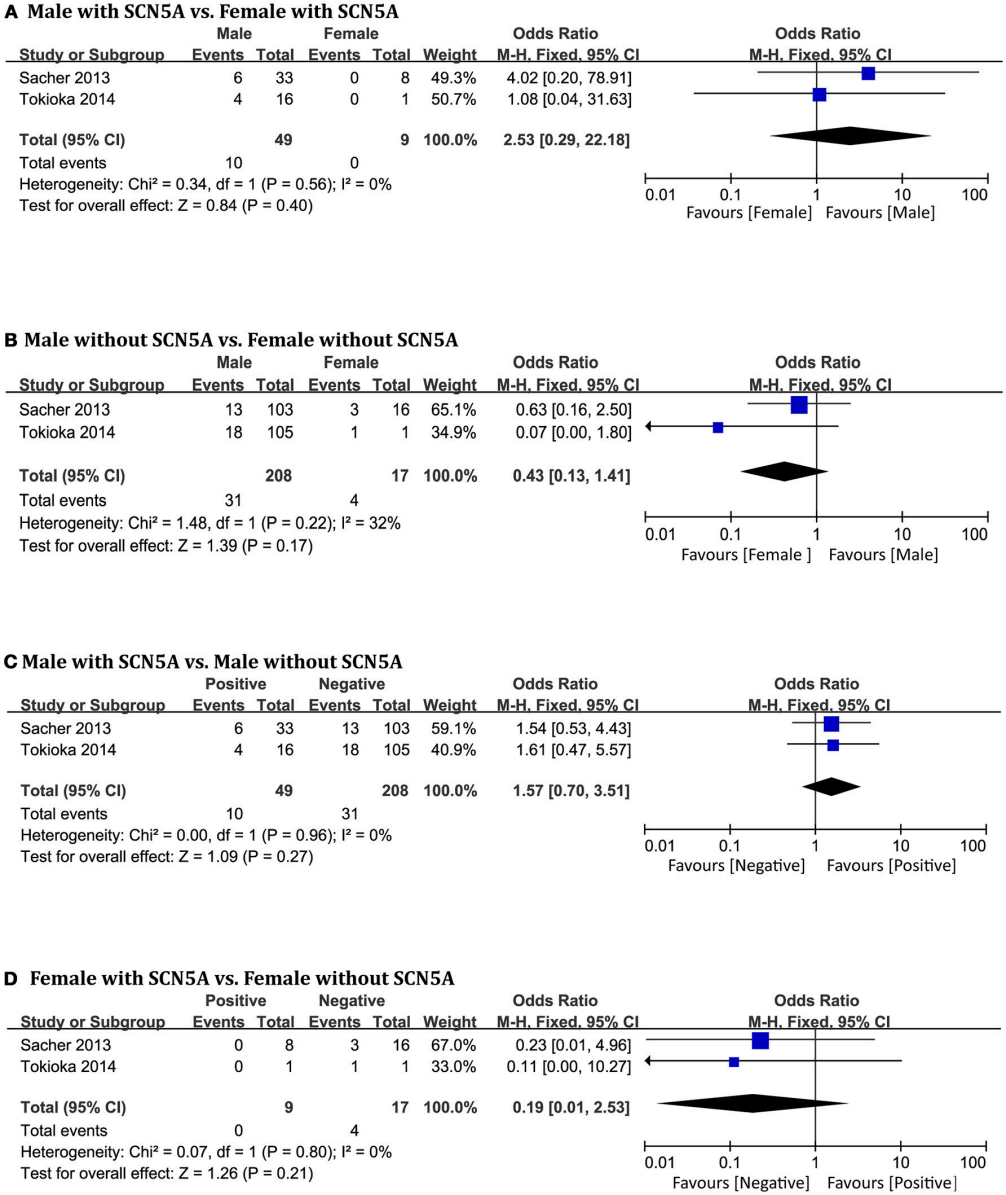
Odds radio for the occurrence of arrhythmic events during follow-up depending on SCN5A pattern subgroups. **(A)** Prognosis of male and female in positive SCN5A subgroup, **(B)** Prognosis of male and female in negative SCN5A subgroup, **(C)** Prognosis of positive SCN5A and negative SCN5A in male subgroup, **(D)** Prognosis of positive SCN5A and negative SCN5A in female subgroup.

Only two original studies (Sacher et al., 2013; Tokioka et al., 2014) including 283 patients (men = 257) were included in this group. In the subgroup positive for *SCN5A* mutations, we found no significant differences related to *SCN5A* between men and women (OR 2.53, 95% CI: 0.29–22.18, *P* = 0.40; heterogeneity: *P* = 0.56, I^2^ = 0%, Figure 6A). In the negative subgroup, the outcome was the same (OR 0.43, 95% CI: 0.13–1.41, *P* = 0.17; heterogeneity: *P* = 0.22, I^2^ = 32%, Figure 6B). No significant differences were found within the male subgroup (OR 1.57, 95% CI: 0.70–3.51, *P* = 0.27; heterogeneity: *P* = 0.96, I^2^ = 0%, Figure 6C), nor within the female subgroup (OR 0.19, 95% CI: 0.01–2.53, *P* = 0.21; heterogeneity: *P* = 0.80, I^2^ = 0%, Figure 6D).

### Symptomatic and asymptomatic

**Figure 7:**
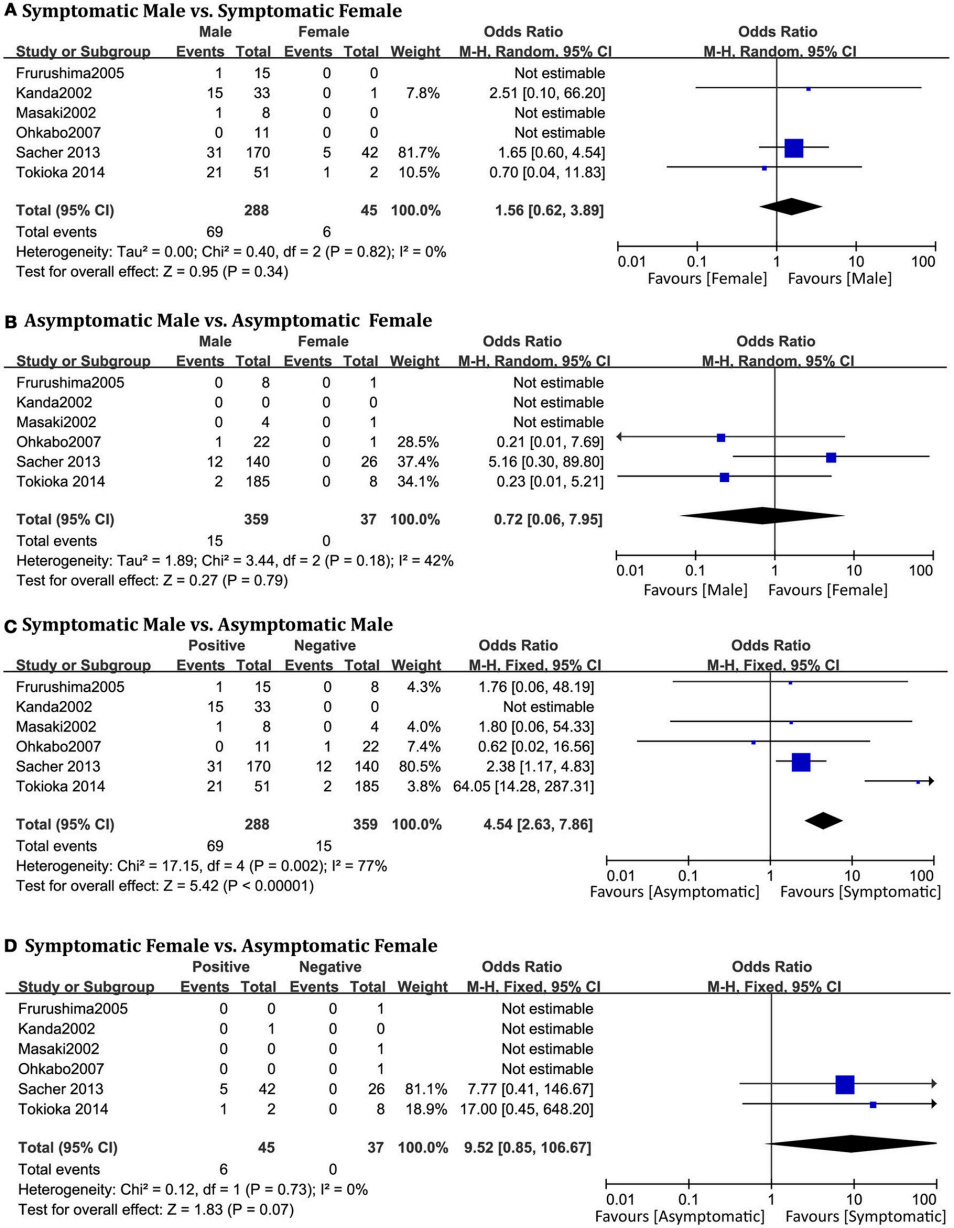
Odds radio for the occurrence of arrhythmic events during follow-up depending on symptomatic pattern subgroups. **(A)** Prognosis of male and female in symptomatic subgroup, **(B)** Prognosis of male and female in asymptomatic subgroup, **(C)** Prognosis of symptomatic and asymptomatic in male subgroup, **(D)** Prognosis of symptomatic and asymptomatic in female subgroup.

**Figure 8:**
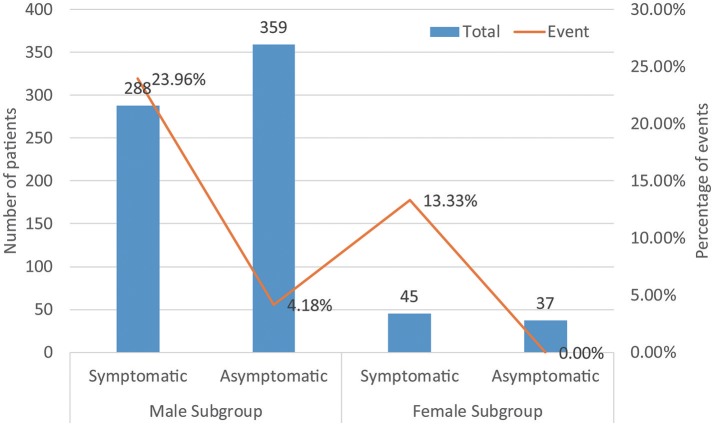
Biaxial diagram depending on number of patients and percentage of events in symptomatic pattern group.

A total of 729 patients (men = 647) in six studies (Kanda et al., 2002; Masaki et al., 2002; Furushima et al., 2005; Ohkubo et al., 2007; Sacher et al., 2013; Tokioka et al., 2014) were eligible for this group. We found that in the male population, symptomatic patients displayed a higher risk of arrhythmic events than asymptomatic patients (OR 4.54, 95% CI: 2.63–7.86, *P* < 0.00001; heterogeneity: *P* = 0.002, I^2^ = 77%, Figure 7C). However, no statistical differences were found within the female population (OR 9.52, 95% CI: 0.85–106.67, *P* = 0.07; heterogeneity: *P* = 0.73, I^2^ = 0%, Figure 7D) (Figure 8). Moreover, there were no significant sex-related differences in the symptomatic subgroup pattern (OR 1.56, 95% CI: 0.62–3.89, *P* = 0.34; heterogeneity: *P* = 0.82, I^2^ = 0%, Figure 7A) or in the asymptomatic subgroup (OR 0.72, 95% CI: 0.06–7.95, *P* = 0.79; heterogeneity: *P* = 0.18, I^2^ = 42%, Figure 7B).

### Documented AF status

**Figure 9:**
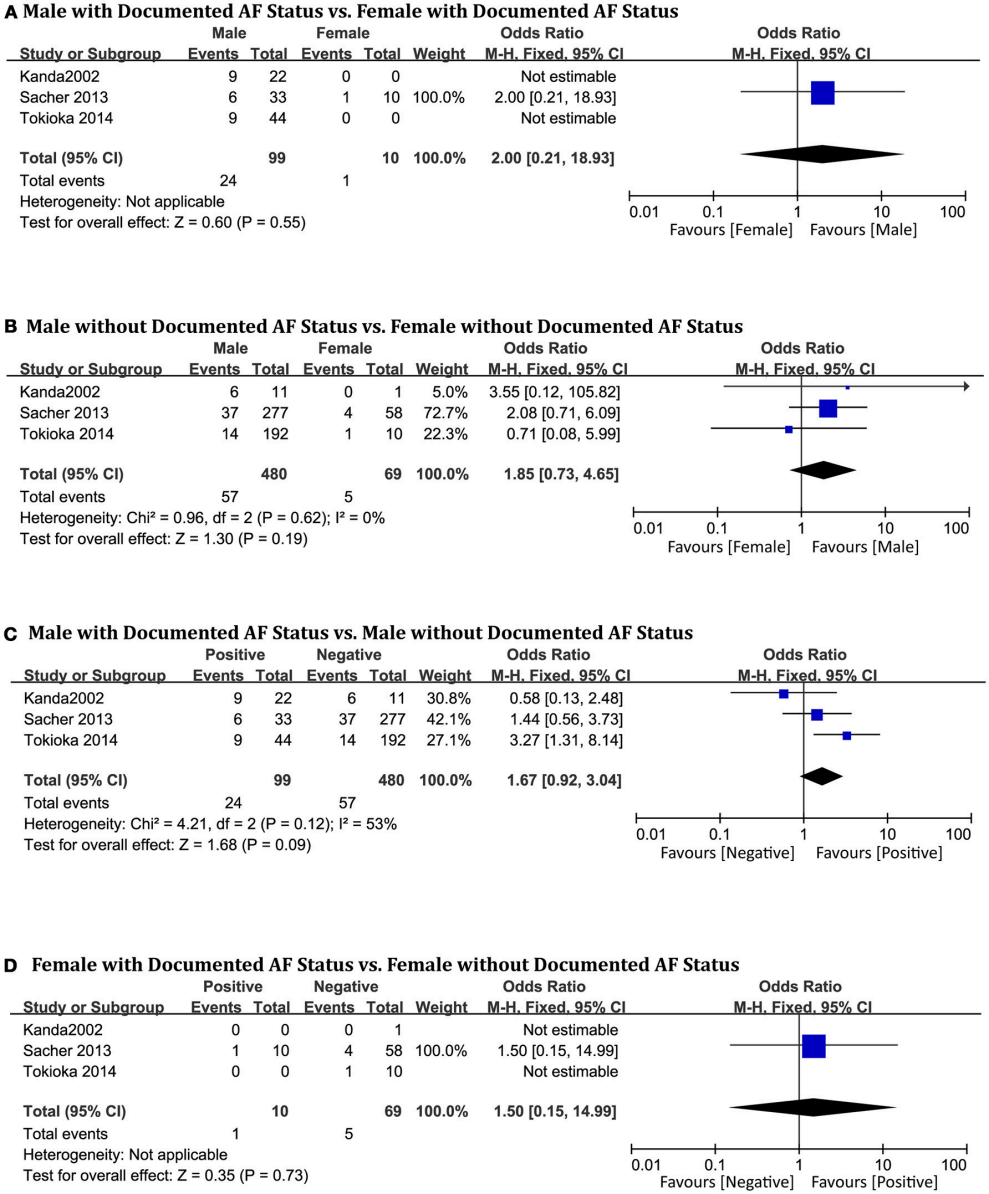
Odds radio for the occurrence of arrhythmic events during follow-up depending on documented AF status subgroups. **(A)** Prognosis of male and female in positive documented AF subgroup, **(B)** Prognosis of male and female in negative documented AF subgroup, **(C)** Prognosis of positive documented AF and negative documented AF in male subgroup, **(D)** Prognosis of positive documented AF and negative documented AF in female subgroup.

The three studies in this analysis (Kanda et al., 2002; Sacher et al., 2013; Tokioka et al., 2014) consisted of 658 patients (men = 579). Sex-related difference was not significantly related to cardiac events in the AF-positive subgroup (OR 2.00, 95% CI: 0.21–18.93, *P* = 0.55, Figure 9A). In the negative group, male and female patients showed no statistical differences (OR 1.85, 95% CI: 0.73–4.65, *P* = 0.19; heterogeneity: *P* = 0.62, I^2^ = 0%, Figure 9B). In the male subgroup, also, there were no significant differences based on documented AF status (OR 1.67, 95% CI: 0.92–3.04, *P* = 0.09; heterogeneity: *P* = 0.12, I^2^ = 53%, Figure 9C). In the female subgroup, the result was the same (OR 1.50, 95% CI: 0.15–14.99, *P* = 0.37, Figure 9D). Heterogeneity was not applicable for some outcomes because only one study provided suitable data for documented AF status.

## Discussion

We drew the following conclusions from the pooled analysis: (i) male patients display a higher risk of arrhythmic events than female patients; (ii) in the male population, symptomatic patients display a higher risk profile of arrhythmic events compared to asymptomatic patients, but there are no significant differences within the female population. Consequently, in the female population, the risk of asymptomatic patterns cannot be underestimated.

According to our systematically comprehensive analysis of 24 trials, male patients display a higher risk profile compared with female patients. Although this conclusion has been consistently recognized in the HRS/EHRA/APHRS expert consensus statement (Priori et al., 2013), our study is the largest at present, including 4,140 patients, to analyze gender differences in prognosis and risk stratification for BrS. Similar outcomes were found in other studies (Gehi et al., 2006; Benito et al., 2008). New studies have confirmed those acknowledged results, and outlined a complex relationship between sex distribution and patient ethnicity and age (Milman et al., 2018).

Many studies have shown that syncope was an independent predictor of risk, and provided sufficient evidence (Brugada et al., 2004; Priori et al., 2012; Calvo et al., 2016). The presence of symptoms in patients was significantly associated with arrhythmic events (23 vs. 3.8%, *P* < 0.00001) in our analysis. These results might explain the conclusion that in the male subgroup, symptomatic patients displayed a higher risk of arrhythmic events than asymptomatic patients. Surprisingly, in the female population, there were no significant differences between symptomatic patients and asymptomatic patients. We can infer that symptomatic status might only be a risk factor for men, and that asymptomatic women may be in a potentially dangerous situation. The risk of asymptomatic patterns cannot be underestimated. Although these results may be due to the lower incidence (11%) of women with BrS, the findings offer new insights for further research to combine with the new syncope episodes (Olde Nordkamp et al., 2015).

In our results, EPS-positive patients had a tendency toward a higher risk of arrhythmic events than EPS-negative patients only in the female subgroup(*p* = 0.06), which presented a potential risk factor to women. We can infer that the result may turn positive when the sample size is enlarged. Whether EPS inducibility is a predictor of arrhythmic events in BrS patients with previous syncope/sudden death or an independent character remains in dispute (Brugada et al., 2002, 2004; Priori et al., 2002; Giustetto et al., 2009). In the 2017 AHA/ACC/HRS guideline for ventricular arrhythmias and SCD, an EPS with programmed ventricular stimulation using single or double extrastimuli may be considered for further risk stratification in asymptomatic and spontaneous type 1patients (Kusumoto et al., 2017). Newly studies suggested that extent of substrate is the only independent predictor of inducibility of VT or VF and may contribute to a new marker for risk stratification and therapy (Pappone et al., 2018). The differences of sex-related cardiac electrophysiological characteristics may be the main reason contributing to the result, that women have lower expression of KChIP2 which is the main accessory subunit of transient outward current in right ventricular epicardium (Tadros et al., 2014). Besides women have greater sinoatrial node automaticity and enhanced atrioventricular node function than men (Burke et al., 1996; Shaowen Liu and Ole Kongstad, 2001).

Spontaneous type 1 ECG was regarded as a risk factor for arrhythmic events in most studies (Brugada and Brugada, 1992; Brugada et al., 1998, 2002, 2004, 2005; Priori et al., 2002, 2012; Benito et al., 2008). Many reporters overserved that men with cardiac events had greater rates of spontaneous type 1 ECG, and among male patients with spontaneous type 1 ECG, cardiac events were more frequent (Benito et al., 2008; Sacher et al., 2013; Shi et al., 2018). Recent studies have indicated that females have less type 1 BrS ECG and lower inducibility rates than males (Milman et al., 2018). However, interestingly no statistically significant sex-related differences were found in our result. In a report by the European Society of Cardiology, family history of SCD is regarded as one of three factors for the events (Priori et al., 2001), but in the family history of the SCD group, we obtained absolutely negative results in all four subgroups. We also observed negative results in the *SCN5A* group, which was consistent with the HRS/EHRA/APHRS expert consensus statement (Priori et al., 2013).

The limitations of the study should be acknowledged. Although we included 4,140 patients from 24 studies incorporating the original data from two articles, there remain limitations in subgroup analysis to a certain extent. The number of women with BrS is relatively small, especially in some small samples. This situation limits the statistical power.

## Author contributions

YG and YX defined the research theme. Dr. Frédéric Sacher and KK contributed the original data. MY and XL wrote the manuscript. XY, YY, and NL designed the methods. CT and XW analyzed the data. HB-M, DH, and HS interpreted the results. All authors discussed the results and commented on the manuscript.

### Conflict of interest statement

The authors declare that the research was conducted in the absence of any commercial or financial relationships that could be construed as a potential conflict of interest.

## Abbreviations

AF: auricular fibrillation
BrS: Brugada Syndrome
ECG: electrocardiogram
EPS: electrophysiological study
ER: early repolarization
f-QRS: fragmented QRS
ICD: implantable cardioverter-defibrillator
RFA: radiofrequency ablation
RVOT: right ventricular outflow tract
SCD: sudden cardiac death
VF: ventricular fibrillation
VT: ventricular tachycardia.
